# Contrast-enhanced Multi-detector CT Examination of Parotid Gland Tumors: Determination of the Most Helpful Scanning Delay for Predicting Histologic Subtypes

**DOI:** 10.5334/jbsr.1596

**Published:** 2019-01-03

**Authors:** Tae-Yeon Kim, Younghen Lee

**Affiliations:** 1 Department of Radiology, Ansan Hospital, Korea, University College of Medicine, Ansan, KR

**Keywords:** Parotid neoplasm, Computed tomography (CT), Contrast media, Image enhancement

## Abstract

**Purpose:**

Although contrast-enhanced CT (computed tomography) is regarded as the preoperative imaging modality of choice for parotid gland tumor, scanning methods are highly variable. We aimed at determining the most helpful scanning delay for predicting histologic subtypes of parotid gland tumors.

**Material and Methods:**

Based on the medical record review, we identified 293 patients with 296 parotid gland tumors who underwent uni- or biphasic neck CT examination using a 64-row detector CT with the same acquisition parameters except the scan delays that were: (1) unenhanced, (2) 40 seconds, (3) 50 seconds, and (4) 70 seconds after the beginning of contrast-media injection. Pathologically, the gland tumors (mean size: 26 ± 10.4 mm) consisted of 164 pleomorphic adenomas, 78 Warthin tumors, 23 other benign tumors, and 31 malignant tumors. The mean CT attenuation values (MAV)s from 419 CT images with different scan delays were compared by analysis of variance (ANOVA).

**Results:**

On enhanced CT with a 50-second scan delay, Warthin tumors most intensely enhanced and could be distinguished from pleomorphic adenomas and malignant tumors (both *p* < 0.05). However, with other scan delays, there were no significant differences in MAV between all histologic subtypes of tumors.

**Conclusion:**

Prediction of histologic subtype, by differentiating Warthin from non-Warthin tumors, was possible only with CT scanning beginning 50 seconds after the start of contrast injection.

## Introduction

Although ultrasound is the first-line imaging tool for salivary gland imaging, it has frequently been combined to contrast-enhanced CT for localizing a deeper lesion and detecting sialolithiasis or metastatic lymph node [1,2,3]. However, the distinction between malignant and benign parotid tumor may be challenging. For example, several subtypes of low-grade parotid malignancies (e.g., mucoepidermoid carcinomas, acinic cell carcinomas, and some adenoid cystic carcinomas) mimic the benign tumors by their smoothly outlined pseudocapsules, in contrast to high-grade malignancies [1,2,3,4,5]. Furthermore, surrounding inflammation and/or hemorrhage of benign parotid gland tumors could mislead to the diagnosis of malignancy. To overcome these considerable morphologic overlaps among the various histologic subtypes of parotid gland tumors, several researchers proposed mono- or multi-phasic contrast enhancement protocols with variable scan delay times, ranging from 30 seconds to 34 minutes [6,7,8,9,10]. However, their studies did not reflect the faster scanning due to technical advances of recent multi-detector computed tomography (MDCT) examination. Moreover, their multi-phasic scanning substantially increases radiation exposure. Therefore, it would be uncertain that aforementioned results might be reproducible at a relatively faster scanning time which does not usually exceed four seconds using advanced scanner with more than 64 rows of detectors.

For differentiating parotid gland tumors, the starting time of CT scanning should be determined to maximize the CT attenuation difference between the different histologic subtypes of parotid gland tumors. To our knowledge, the need for scan protocol optimization that could significantly alter the degree of tissue enhancement was overlooked in the recent literature demonstrating the superior diagnostic performance of textural analysis of multi-energy datasets to classify benign parotid gland tumors [11]. In this study, we determined the scan delay of contrast-enhanced CT protocol relevant to predict histologic subtypes of parotid gland tumors, using our 64-row detector neck CT protocols with otherwise identical injection and acquisition parameters.

## Material and Methods

### Patients

This study used retrospectively collected medical records from three hospitals. Approval to perform this study was obtained from our institutional review board (IRB number: K2018-0790-001), and the requirement for informed patient consent was waived.

Based on a retrospective search through three university hospitals under our medical center, we found that some patients with suspicious salivary gland tumor were preoperatively examined by the same MDCT scanner from July 2008 to June 2010. During this period, 311 patients underwent superficial or total parotidectomy to remove tumors. Among them, injection and acquisition parameters of 293 patients (166 females, 127 males; mean age: 51.2 years; age range: 19–83 years) who were evaluated by the same type of MDCT scanner (Brilliance 64-row detector CT; Philips Medical Systems, Cleveland, OH, USA) were identical, except the total number of scanning and scan delay that could alter the degree of contrast enhancement. Their pathologic reports revealed three patients with bilateral tumors and 290 patients with a single tumor. Thus, a total of 296 pathologically confirmed parotid gland tumors in 293 patients were finally analysed. Out of those 296 parotid tumors, 143 were on the right side and 153 on the left. The maximum diameters of the tumors ranged from 0.8 to 5.7 cm (mean 2.6 ± 0.7 cm).

We classified all parotid gland tumors into four subtypes, based on the histologic results: 164 pleomorphic adenomas, 78 Warthin tumors, 23 other benign tumors (15 basal cell adenomas, 2 canalicular adenomas, 3 monomorphic adenomas, and 3 oncocytomas), and 31 malignant tumors (15 mucoepidermoid carcinomas, 4 squamous cell carcinomas, 4 acinic cell carcinomas, 3 salivary duct carcinomas, 2 adenoid cystic carcinomas, 1 adenocarcinoma, 1 carcinoma ex pleomorphic adenoma, and 1 carcinosarcoma). There was no statistical significant difference among the four classified types of parotid gland tumors in relation to the maximum diameter. None of them had any history of acute or chronic inflammation nor previous treatments over the parotid region, such as radiation, which could alter the parotid parenchymal CT attenuation.

### Contrast-enhanced MDCT Image Acquisition

Since the installation of the same 64-row MDCT scanners, CT acquisition protocols for salivary gland tumors were identical among our three hospitals between July 2008 and June 2010, except the total number of scanning and scan delay.

The enrolled patients were scanned using the following settings: rotation time, 0.5 seconds; beam collimation, 64 × 0.625 mm; helical pitch (beam pitch), 0.89; table movement, 71.3 mm/s; tube voltage, 120 kVp; tube current, 250 mAs; and field of view, 220 mm. All axial and coronal images were reconstructed in a soft-tissue window setting (window level and width: 60 HU [Hounsfield units] and 300 HU, respectively), every 3 mm on a 512 × 512 matrix. In each patient, the scan delay was defined as the time from the beginning of contrast infusion to that of CT data acquisition. Scanning started at the base of skull and continued toward the aortopulmonic window, once or twice with the same maneuver, before contrast infusion or 40, 50, or 70 seconds after the onset of contrast infusion.

For contrast-enhanced images, a fixed total dose of 80 mL of nonionic iodinated contrast agent (Ultravist 300 mgI/mL, Bayer-Schering Pharma, Berlin, Germany) was administered into an antecubital vein at a rate of 2.5 mL/s using a power injector (MCT; Medrad, Pittsburgh, PA, USA) via a 20-gauge intravenous catheter. Immediately after a contrast infusion, 20 mL of a normal saline solution was injected at a rate of 3 mL/s to reduce the perivenous artifacts, induced by stagnant contrast within the subclavian vein [12]. The right arm was preferentially selected whenever possible to avoid delayed contrast arrival by physiologic compression of the left brachiocephalic vein as a potential problem if the left arm was used [12].

### Image Analysis

One neuroradiologist (with 10 years of post-training experience in head and neck radiology), who was blinded to histologic results of parotid gland tumors, measured the mean attenuation values (MAV)s by drawing circular regions-of-interest (ROIs) cursors over the area of each parotid tumor on the different scanning time delay images: (1) unenhanced, (2) 40 seconds, (3) 50 seconds, and (4) 70 seconds after beginning of contrast infusion. The ROI circles were made as large as possible in the representative portion of the parotid gland tumors with sufficient margin to avoid beam hardening artifact from an adjacent mandible or metallic dental prosthesis. In addition, the MAVs for parotid parenchyma were measured in ipsilateral, homogeneous regions devoid of parotid tumor or prominent artifacts by maintaining a constant ROI area of approximately 100.0 mm^2^.

### Statistical Analysis

The statistical analysis was performed using commercially available software (SPSS for Windows version 20.0, IBM Corp.). To determine which of the following scan delays might be helpful to predict the histologic subtypes of parotid gland tumors, we compared the MAVs of all tumors obtained depending on the different scan delays including unenhanced images, using a one-way analysis of variance (ANOVA). When the overall differences between the unenhanced and contrast-enhanced images with three different scan delays were statistically significant, Bonferroni post-hoc analysis was performed for multiple comparisons to examine particular differences. A value of *p* < 0.05 was considered to indicate a statistically significant difference.

## Results

### Tumor Characteristics According to Different Scan Delays

Of all the 296 parotid gland tumors, 173 were evaluated by a monophasic scanning: (1) unenhanced: 63 cases, (2) 40-second scan delay: 9 cases, (3) 50-second scan delay: 94 cases, (4) 70-second scan delay: 7 cases; 123 were evaluated by biphasic scanning: (1) unenhanced and 40-second scan delay: 6 cases, (2) unenhanced and 50-second scan delay: 17 cases, (3) unenhanced and 70-second scan delay: 26 cases, (4) 40-second and 70-second scan delay: 74 cases. Finally, the numbers of images allocated into unenhanced, 40-second, 50-second, and 70-second scan delay ranged from 89 to 112 (Table 1). There were no significant differences in distribution of the patient’s age and gender, the maximum diameter of the parotid gland tumors, and pathologic diagnosis according to the scanning delay (all *p* > 0.05).

**Table 1:** Tumor Characteristics.

Characteristics	Scan delay				

	Unenhanced	40-s	50-s	70-s	Total

No. of CT examination*	112	89	111	107	419
No. of pathologic diagnosis (P:W:O:M)*	73:20:11:8	46:21:12:10	59:38:0:14	53:26:19:9	196:95:33:35
Sex ratio (M:F)*	56:56	48:41	49:62	55:52	208:211
Age, Mean ± SD (Y)*	46.3 ± 15.0	46.5 ± 15.9	48.5 ± 17.1	45.8 ± 14.0	48.2 ± 15.2
Size of parotid tumor, Mean ± SD (cm)*	2.5 ± 0.8	2.5 ± 1.0	2.9 ± 1.1	2.3 ± 0.9	2.3 ± 0.9

*Note*: SD: standard deviation, P: pleomorphic adenoma, W: Warthin tumor, O: other benign tumor, M: malignant tumor. *No significant difference was found between unenhanced, 40-s, 50-s, and 70-s scan delay.

### Effect of Scan Delay on MAVs of Parotid Parenchyma, Parotid Gland Tumors with Different Histologic Subtypes

The MAVs of parotid gland tumors and ipsilateral parotid parenchyma on unenhanced and enhanced CT images with different scan delays are presented in Table 2. The MAVs of ipsilateral parotid glands for unenhanced, 40-second, 50-second, and 70-second protocols were –9.99 ± 23.84, 18.81 ± 19.67, 21.38 ± 17.88, and 24.12 ± 21.62 HU, respectively. The MAVs of parotid parenchyma obtained from all contrast-enhanced CT images were significantly higher as compared to those of the unenhanced CT images (*p* < 0.001). However, the differences between MAVs of parotid parenchyma were not statistically significant among the contrast-enhanced CT images with 40 seconds, 50-seconds, and 70 seconds (*p* > 0.05).

**Table 2:** MAVs (HU) of Parotid Gland Tumors and Ipsilateral Parotid Parenchyma Obtained at Unenhanced and Enhanced Images with Different Scan Delay.

	Parotid parenchyma	P	W	O	M

Unenhanced (total N =112, P:W:O:M = 73:20:11:8)	–9.99 ± 23.84	31.29 ± 15.36	37.28 ± 11.69	34.98 ± 20.87	38.05 ± 20.66
40-s (total N = 89, P:W:O:M = 46:21:12:10)	18.81 ± 19.67	59.50 ± 27.71	78.13 ± 19.65	63.62 ± 21.71	66.91 ± 31.95
50-s (total N = 111, P:W:O:M = 59:38:0:14)	21.38 ± 17.88	67.04 ± 25.52	102.09 ± 21.78	NA	74.15 ± 20.23
70-s (total N = 107, P:W:O:M = 53:26:19:9)	24.12 ± 21.62	69.98 ± 27.65	70.36 ± 23.92	71.50 ± 21.67	65.49 ± 28.84

*Note*: Based on the mean ± standard deviation, P: pleomorphic adenoma, W: Warthin tumor, O: other benign tumor, M: malignant tumor, NA: Not applicable.

**Figure 1:**
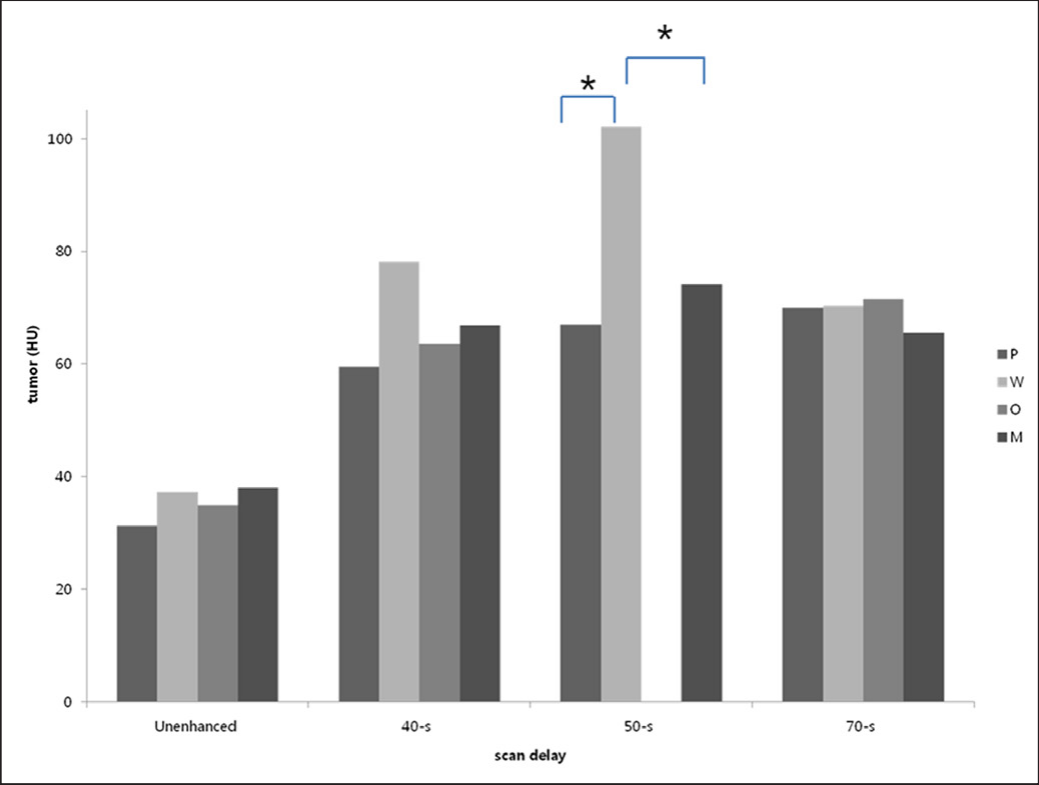
Graphs show a comparison of the MAVs of parotid gland tumors on the unenhanced and enhanced images, with different scan delay. Except the differences of the MAVs between Warthin tumors and pleomorphic adenoma or malignant tumors on the images at a 50-second scan delay, there was no statistically significant differences among the different histologic subtypes of parotid gland tumors (*p < 0.05).

The MAVs of malignant parotid gland tumors were not distinguishable from those of all other benign tumors (*p* > 0.05), except for Warthin tumors on the contrast-enhanced CT images with only 50-second scan delay (Table 3, Figure 1). The MAVs of malignant tumors versus warthin tumors on 50-second delay scans, were 74.15 ± 20.23 versus 102.09 ± 21.78 HU (*p* < 0.05).

**Table 3:** Post-hoc Tests of MAVs Obtained from Parotid Gland Tumors with Different Histologic Subtypes on Each Scan Delay.

Scan delay	Histologic subtypes (I)	Histologic subtypes (J)	Mean difference (I-J)	Standard Error	P value	95% confidence interval	

						Lower Bound	Upper Bound

unenhanced	P	W	–8.94	15.55	1.000	–50.4	32.5
		O	–3.69	5.40	0.98	–20.26	12.87
		M	–9.76	4.82	0.32	–25.39	5.87
	W	O	9.72	5.72	0.47	–7.35	26.78
		M	3.65	5.18	0.98	–12.38	19.67
	O	M	–6.07	6.78	0.93	–26.05	13.92

40-s	P	W	–10.63	10.47	0.89	–39.90	18.63
		O	18.87	20.04	0.91	–49.96	87.70
		M	3.59	16.39	1.00	–51.11	58.28
	W	O	29.50	20.74	0.65	–39.46	98.47
		M	14.22	17.24	0.95	–41.13	69.57
	O	M	–15.28	24.27	0.99	–91.04	60.47

50-s	P	W	–25.04	5.14	0.00	–37.53	–12.55
		O	NA	NA	NA	NA	NA
		M	–7.11	6.81	0.66	–24.28	10.06
	W	O	NA	NA	NA	NA	NA
		M	17.93	6.21	0.03	1.94	33.92
	O	M	NA	NA	NA	NA	NA

70-s	P	W	–.38	6.96	1.00	–19.46	18.70
		O	–9.51	12.29	0.96	–45.63	26.62
		M	4.49	12.64	1.00	–40.36	49.34
	W	O	–9.13	12.53	0.97	–45.73	27.47
		M	4.87	12.88	0.99	–39.90	49.64
	O	M	–13.99	16.38	0.94	–63.50	35.51

*Note*: P: pleomorphic adenoma, W: Warthin tumor, O: other benign tumor, M: malignant tumor, NA: Not applicable. Adjustment for multiple comparisons: Bonferroni.

**Figure 2:**
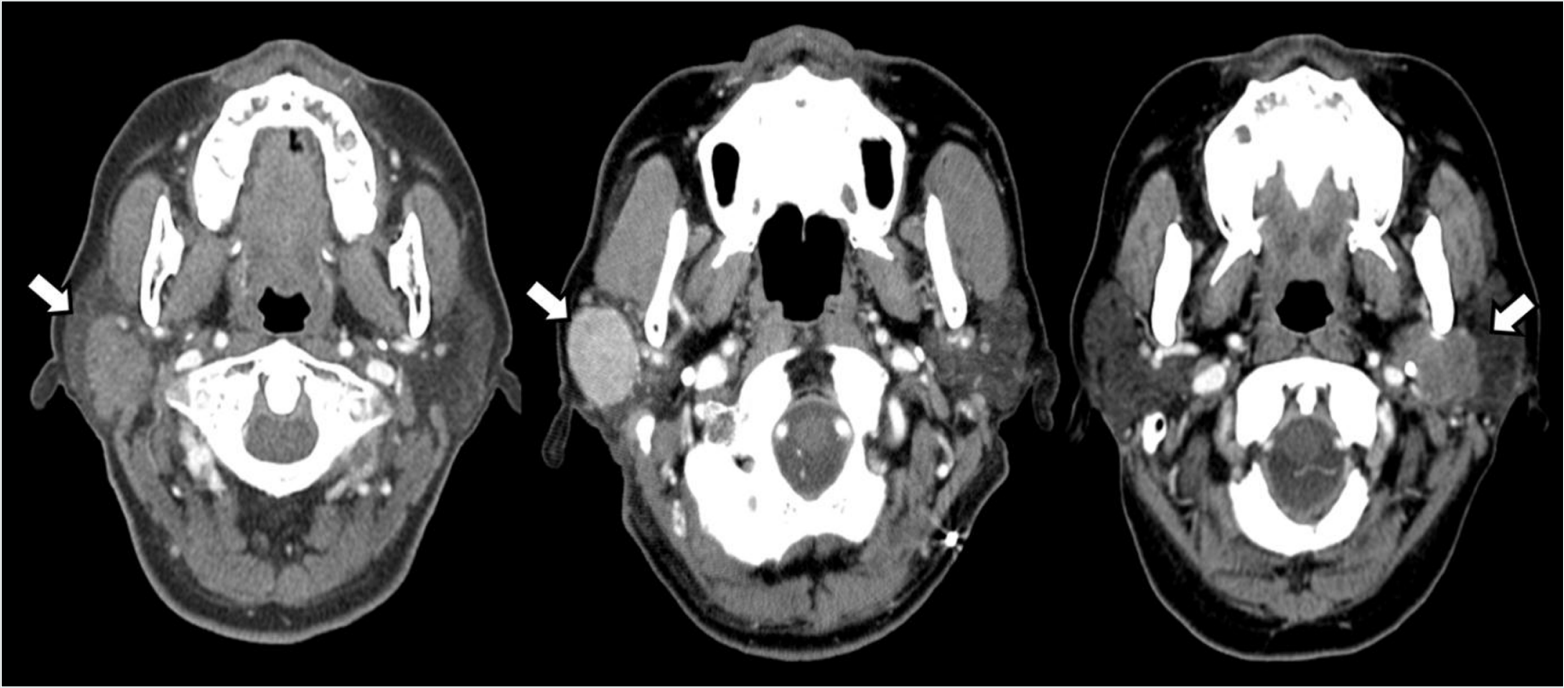
Contrast-enhanced CT images of parotid gland tumors (arrows) in three different patients (the left, 57-year-old man; the center, 71-year-old man; the right, 44-year-old woman) obtained 50 seconds after beginning of contrast infusion. Warthin tumor (center) shows stronger enhancement compared to pleomorphic adenoma (left) and mucoepidermoid carcinoma (right).

**Figure 3:**
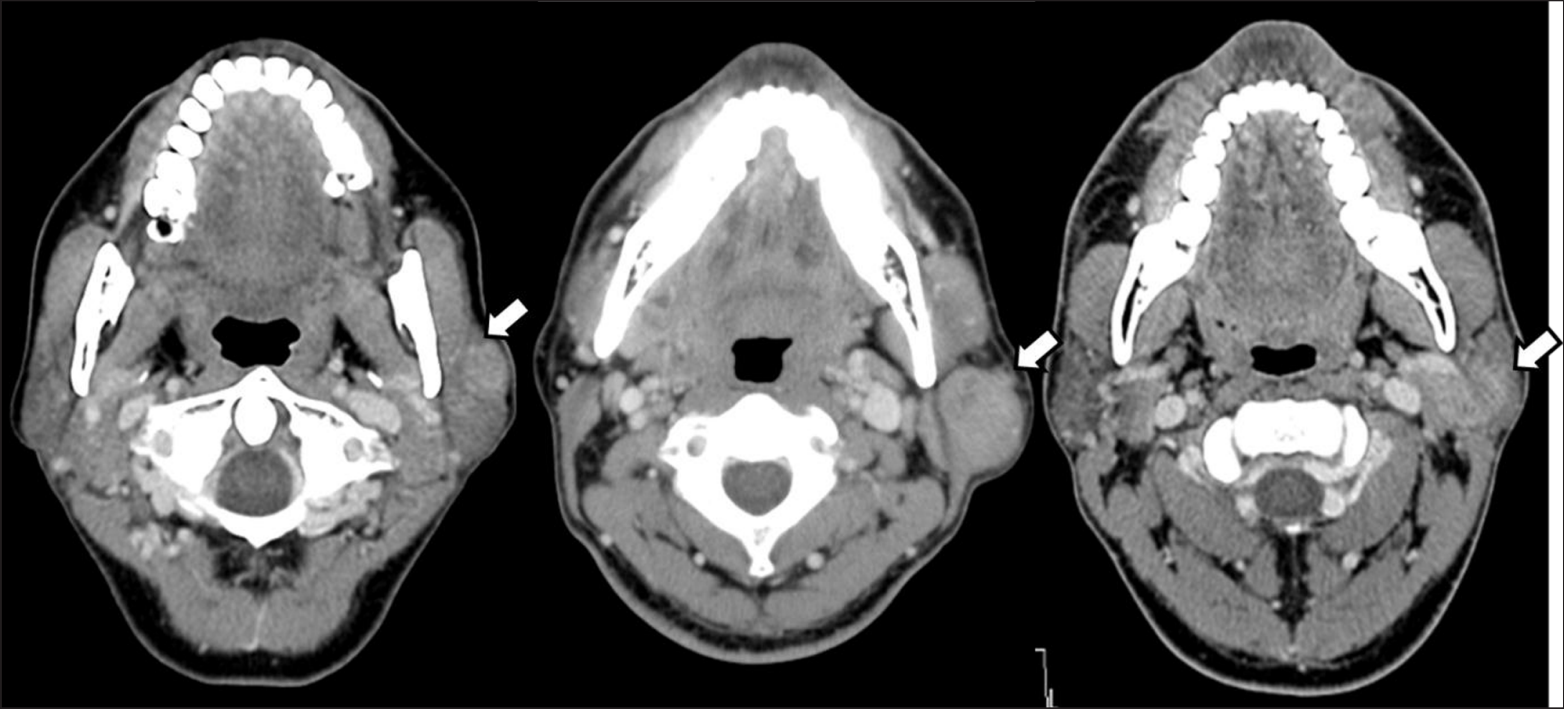
Contrast-enhanced CT images of parotid gland tumors (arrows) in three different patients (the left, 49-year-old woman; the center, 45-year-old man; the right, 68-year-old man) obtained 70 seconds after beginning of contrast infusion. As compared with a 50-second scan delay, Warthin tumor (center) does not show strong enhancement, a characteristic finding to suggest rich microvessels, it is therefore hardly distinguished from other tumors (pleomorphic adenoma [left] and acinic cell carcinoma [right]).

Moreover, on contrast-enhanced images obtained at only 50-second scan delay, MAVs of Warthin tumors, 102.09 ± 21.78 HU, were significantly higher than those of pleomorphic adenomas, 67.04 ± 25.52 HU (*p* < 0.01) (Figure 2); whereas on 70-second scan delays, those of Warthin tumors dropped to 70.36 ± 23.92 HU (Figure 3).

## Discussion

From our quantitative measurement, significant differences did not exist simultaneously between the malignant and benign tumor groups. Early scanning protocol, such as 50-second delay, might be more helpful to maximize the CT attenuation difference from Warthin from non-Warthin tumors, as the more delayed contrast-enhanced images, such as 70-second scan delay, were less helpful.

Like in other organs, the contrast-enhancement features of parotid gland tumors are influenced by their microvasculature, cellularity, and stromal component. Warthin tumors, known as the second most common benign parotid gland tumors, have rich microvasculature [6,9,13]. Accordingly, the significant difference of CT attenuation between pleomorphic adenomas and Warthin tumors observed at a 50-second scan delay could be explained by peak enhancement of Warthin tumors, which was similar to the previous study of Choi et al., who first described a pattern of strong enhancement on early scanning (30 seconds) with a washout on delayed scanning (120 seconds) [9]. On the other hand, pleomorphic adenomas, as the most common type of parotid gland tumors, showed a gradual MAV increase on 40- to 70-second scan delay CT images, similar to that of malignant parotid gland tumors. Such a slow infusion of contrast media in these tumors is likely due to their abundant myxoid or fibrous stromal elements, which retain contrast media in the late phases [4,5,6,14]. Furthermore, pleomorphic adenomas contain varying proportions of epithelial and myxoid elements with frequent “metaplastic” differentiation into oncocytic, sebaceous, mucinous, squamous, chondroid, osseous, or adipose cells, so that their histologic features are highly heterogeneous, even within the same tumor. Accordingly, our results were consistent with the previous studies highlighting this heterogeneity [4,5,14,15,16].

This study has a number of limitations. First, it did not assess CT images with more prolonged (>70-second scan delay). Therefore, our protocols failed to provide sufficient data to obtain the time-attenuation curves for individual subtypes of parotid gland tumors. Secondly, although we suggested that the contrast-enhanced CT images with a 50-second scan delay might be more helpful to differentiate Warthin tumors from non-Warthin tumors, we did not provide a reliable criterion, such as CT attenuation value, to define strong enhancement. In addition to the scan delay, the injection parameters and the patient’s condition, including concentration, total amount, flow rate of iodinated contrast, and patient’s cardiac output, all of which could substantially affect the degree of contrast enhancement, still remain highly variable. Thus, the reference values based on the present protocol might not be reproducible in different conditions.

In summary, our MDCT contrast enhancement protocol with different scan delay ranging from 40 to 70 seconds revealed that prediction of histologic subtypes of parotid gland tumors was only possible on the CT images obtained with a 50-second scan delay by strongly discriminating Warthin from non-Warthin tumors, instead of malignant versus benign tumors.
